# Phylodynamics of Highly Pathogenic Avian Influenza A(H5N1) Virus Circulating in Indonesian Poultry

**DOI:** 10.3390/v14102216

**Published:** 2022-10-08

**Authors:** Desniwaty Karo-karo, Rogier Bodewes, Restuadi Restuadi, Alex Bossers, Agustiningsih Agustiningsih, Jan Arend Stegeman, Guus Koch, David Handojo Muljono

**Affiliations:** 1 Department Population Health Sciences, Faculty of Veterinary Medicine, Utrecht University, 3584 CL Utrecht, The Netherlands; 2 Centre of Diagnostic Standard Indonesian Agricultural Quarantine Agency, Ministry of Agriculture, Jakarta 13220, Indonesia; 3 National Institute for Public Health and the Environment, 3720 BA Bilthoven, The Netherlands; 4 Great Ormond Street Institute of Child Health, University College London, London WC1N 1EH, UK; 5 Institute for Risk Assessment Sciences (IRAS), Department Population Health Sciences, Faculty of Veterinary Medicine, Utrecht University, 3584 CL Utrecht, The Netherlands; 6 National Agency for Research and Innovation of The Republic of Indonesia, Jakarta 10340, Indonesia; 7 Wageningen Bioveterinary Research, 8221 RA Lelystad, The Netherlands; 8 Faculty of Medicine, Universitas Hasanuddin, Makassar 90245, Indonesia; 9 Faculty of Medicine and Health, University of Sydney, Camperdown, NSW 2006, Australia; 10 Eijkman Institute for Molecular Biology, Jakarta 10430, Indonesia

**Keywords:** HPAI, H5N1, Indonesia, phylodynamic, Bayesian evolutionary analysis

## Abstract

After its first detection in 1996, the highly pathogenic avian influenza A(H5Nx) virus has spread extensively worldwide. HPAIv A(H5N1) was first detected in Indonesia in 2003 and has been endemic in poultry in this country ever since. However, Indonesia has limited information related to the phylodynamics of HPAIv A(H5N1) in poultry. The present study aimed to increase the understanding of the evolution and temporal dynamics of HPAIv H5N1 in Indonesian poultry between 2003 and 2016. To this end, HPAIv A(H5N1) hemagglutinin sequences of viruses collected from 2003 to 2016 were analyzed using Bayesian evolutionary analysis sampling trees. Results indicated that the common ancestor of Indonesian poultry HPAIv H5N1 arose approximately five years after the common ancestor worldwide of HPAI A(H5Nx). In addition, this study indicated that only two introductions of HPAIv A(H5N1) occurred, after which these viruses continued to evolve due to extensive spread among poultry. Furthermore, this study revealed the divergence of H5N1 clade 2.3.2.1c from H5N1 clade 2.3.2.1b. Both clades 2.3.2.1c and 2.3.2.1b share a common ancestor, clade 1, suggesting that clade 2.3.2.1 originated and diverged from China and other Asian countries. Since there was limited sequence and surveillance data for the HPAIv A(H5N1) from wild birds in Indonesia, the exact role of wild birds in the spread of HPAIv in Indonesia is currently unknown. The evolutionary dynamics of the Indonesian HPAIv A(H5N1) highlight the importance of continuing and improved genomic surveillance and adequate control measures in the different regions of both the poultry and wild birds. Spatial genomic surveillance is useful to take adequate control measures. Therefore, it will help to prevent the future evolution of HPAI A(H5N1) and pandemic threats.

## 1. Introduction

In 1996, the first outbreak of highly pathogenic avian influenza virus (HPAIv) A(H5N1) occurred in China. Subsequently, this virus from the goose/Guangdong (Gs/Gd) lineage spread to multiple other countries. Nowadays, outbreaks of HPAIv A(H5N1) and related HPAIv have caused economic losses due to the deaths and culling of millions of chickens and other poultry worldwide. In addition, 865 human cases of HPAIv A(H5N1) infections were reported with a case-fatality rate of 53% from 2003 to 2022 [1].

The HPAIv A(H5N1) virus was first reported in Indonesia in 2003 and became endemic in multiple regions afterward. The introduction to and spread of HPAIv A(H5N1) within Indonesia was facilitated by several factors [2]. First, Indonesia is located at the crossroads of international trade between two continents (Asia and Australia) and two oceans (Peace, the Indian Oceans). Second, two wild bird migratory flyways, the East Asian–Australasian (EAAF) and the West Pacific (WPF) flyways include Indonesia. Third, the high contact rate between poultry from different locations [3] and between domestic ducks and wild birds due to poor biosecurity, particularly for backyard and moving or scavenging ducks [4]. Virus transmission between farms was facilitated by poultry trade and live bird markets and by human–animal interaction from inbound and outbound visits to poultry farms and live bird markets. Humans, via contact with poultry, could act as a vector of HPAIv A(H5N1) and facilitate transmission between poultry flocks [3,5]. 

Molecular surveillance is an important tool to support the control of HPAIv A(H5N1). HPAI genome sequence data obtained from avian and human cases can be used to understand transmission pathways [6], identify molecular markers for disease [7,8], expand host coverage [9], and detect variants associated with vaccine escape [10]. Molecular surveillance can also help to identify possible genetic drift and reassortments of HPAIv A(H5N1) with other influenza A viruses that may result in newly emerging viruses with possible increased transmission in poultry and wild birds, different pathogenicity which may also result in a wider host range [11,12]. 

Based on the global analysis of genomic data of HPAIv A(H5N1) detected in Indonesia, HPAIv A (H5N1) were classified into various clades, starting with clade 2.1, which subsequently branched into clades 2.1.1, 2.1.2, 2.1.3.2, and 2.1.3.2a; most clades have been reported to affect poultry [13,14,15]. In 2012, a new clade, 2.3.2.1c, was isolated from a duck farm and live bird markets in Java with high mortality among duck and amino changes such as a Ser deletion at position 325 in the multibasic amino acid cleavage site, and a K328R substitution [16]. The detection of HPAIv from this new clade was thought to be the result of a new incursion from other parts of South East Asia to Indonesia [11,17], as the clades 2.3.2.1, 2.3.2.1a, and 2.3.2.1b have been reported in other South-East countries such as China, Vietnam and Bangladesh [13,14,18,19]. Clade 2.3.2.1c subsequently circulated in poultry, while HPAIv from clade 2.1.3.2a was only detected in Sumatra [11,16,20,21,22]. A molecular study of HPAIv A(H5N1) carried out in 2015 and 2016 suggested that this new clade had diverged into two putative subgroups, clades 2.3.2.1c (A) and 2.3.2.1c (B) [11]. 

Although the major clades of HPAIv A(H5N1) in Indonesia are known, there is limited understanding of the evolution of HPAIv A(H5N1) in Indonesia. This knowledge can be useful to help focus surveillance and strengthen control measures aiming to reduce future reassortments and transmission of HPAIv among poultry and humans. The present study aimed to increase the knowledge of HPAIv A(H5N1) evolution in Indonesia from 2003–2016, with a particular focus on the HA gene segment and the jump of the clades of H5N1v.

To this end, we analyzed the available sequences of hemagglutinin (HA) in the genome database to improve the understanding of the phylodynamics of HPAIv A(H5N1) in Indonesia.

## 2. Materials and Methods

### 2.1. Dataset Preparation

Complete sequences of HA genes obtained from HPAIV A(H5Nx) detected in poultry in Indonesia from 2003 to 2016 were downloaded from the genome database, GISAID, and GENBANK and compiled as Indonesian H5N1 (HA). Another data compilation was downloaded from all available global sequences including Indonesia from 1966 to 2022 and separated as Global H5 (HA). Additional separated data for clades 2.3.2.1c, 2.3.2.1a, and 2.3.2.1 were also downloaded from the database. The HA gene was chosen because the HA protein is located on the outer surface of the virus particle, has a role in the virus–host cell interaction and is the main target for the protective antibody response [23]. Additionally, HA genes are published most frequently in the genome database, indicating that a worldwide phylodynamic analysis of H5N1v using HA genes will provide the most information. 

The sequences were then aligned using MUSCLE [24] and the HA clades of the virus were phylogenetically analyzed using MEGA 7 [25] as described in a previous study [11]. The clade of HA was confirmed using the Highly Pathogenic H5N1 Clade Classification Tool of the Influenza Research Database (https://www.fludb.org/brc/home.spg?decorator=influenza, last accessed on 13 September 2022). 

### 2.2. Clustering HA Gene Segments

The dataset of HA genome sequences was processed with cd-hit-est software of the CD-HIT Suite (http://weizhong-lab.ucsd.edu/cdhit_suite/cgi-bin/index.cgi?cmd=cd-hit-est, last accessed on 13 September 2022) to cluster sequences that shared 100% nucleotide identity [26,27,28]. The CD-HIT_EST test was performed on the globally available 12,018 HA genome sequences (1966–2022) irrespective of the accompanying NA. To condense the global taxa of the full genomes of HA genes, 80 to 99% identity thresholds were examined to obtain the cluster representative sequences. Maximum-likelihood analysis with bootstrapping was performed at different thresholds, and clusters of representative taxa were selected from taxa that share a larger identity than 98%. The representative sequences were used as a dataset for time-scale phylogeny analysis and demography reconstruction. 

### 2.3. Time-Scale Phylogeny of Indonesian HPAIv A(H5N1) Sequences

Divergence times and evolutionary analysis were estimated simultaneously with Bayesian phylogenetic inference (BI) implemented in BEAST v.2.6.7 [29] (http://www.beast2.org/). The optimal substitution model was selected by the BEAST-ModelTest (bModelTest) v.1.2.1 package implemented in BEAST using transdimensional Markov chain Monte Carlo (MCMC) methods [30]. The best substitution model from bModelTest was also compared to the best substitution model selected by the Modeltest in the phangorn package implemented in the R (version R-4.0.3) environment for statistical analysis. bModelTest was also used to infer the gamma-distributed rate of heterogeneity, invariable site proportions, and unequal base frequencies [30]. 

Tree and clock priors were set on a coalescent Bayesian skyline tree and a relaxed molecular clock (assuming an uncorrelated lognormal distribution clock model) which was calibrated by using the sample collection dates. The Bayesian MCMC analysis was performed for 150–300 million generations sampled every 1000–3000 generations. 

The parameter convergences were viewed and evaluated using Tracer v.1.7.1 [31] (http://tree.bio.ed.ac.uk/software/tracer/). The maximum clade credibility (MCC) phylogenetic trees were constructed by TreeAnnotator v.2.6.7 (BEAST package) by removing the initial 10–25% (burn-in) trees (burn-in settings depend on convergence). Then, phylogenetic trees were visualized by using FigTree version 1.4.4 (http://tree.bio.ed.ac.uk/software/figtree/). The calendar date of origin of tMRCA of Indonesian HPAIv A(H5N1) estimated in the BEAST analysis was converted using the lubridate package (https://lubridate.tidyverse.org/) implemented in R (version R-4.0.3). 

The first step of BEAST was to analyze the Indonesian HA HPAIv A(H5N1) gene segments from viruses collected from 2010 to 2016. Then, HA gene segment analysis was performed separately on viral sequences of three different HA clades, clades 2.1.3.2, 2.1.3.2a, and 2.3.2.1c, collected from 2005 to 2016. To confirm the evolution in 2010–2016, MCMC analysis (BEAST) of the Indonesian HA HPAIv A(H5N1) gene was performed over the extended period of 2003–2016. The BEAST analysis over the period 2003–2016 is displayed in the results of Indonesian HA (H5).

In the final stage, we performed BEAST to analyze the worldwide HA of all available avian influenza viral sequences (2005–2021). The full length 12,018 HA sequences were downloaded from GISAID and clustered using CD-HIT-EST as described above. the Reference sequences closely related to Indonesian HPAIv H5N1 according to the maximum likelihood tree were selected and aligned using MEGA 7 [25] before proceeding to the BEAST analysis. 

## 3. Results

### 3.1. Bayesian Evolutionary Analysis of HA of Indonesian H5N1 

The number of taxa used for the BEAST analysis performed on HA sequences from Indonesia and worldwide with different times of collection and the number of sites is presented in Table 1.

Time-measured phylogenetic analysis of 1707 sites from 94 taxa using the substitution model TIM1 + Γ + I showed the evolution of various clades HPAIv A(H5N1) in Indonesia (Table 1). 

**Figure 1:**
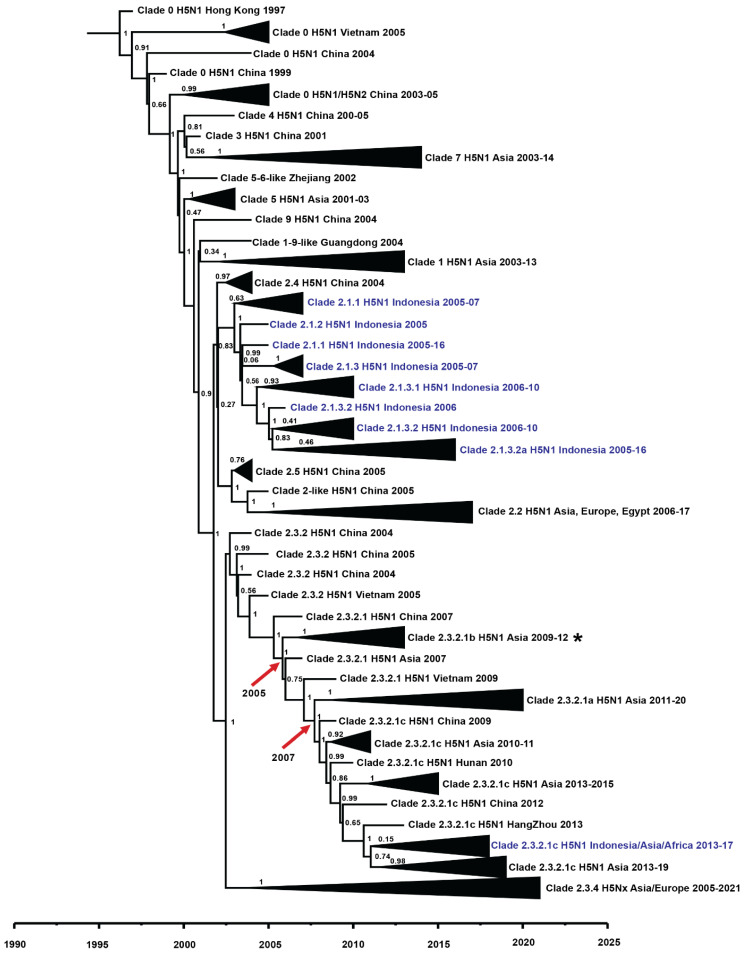
Time-scale phylogeny of selected worldwide HA of H5N1v. The estimated origin of the divergence of the HA 2.3.2.1c clade is highlighted in the asterisk symbol. The tMRCA of HA clades 2.3.2.1b and 2.2.3.1a are pointed out by the arrow. The blue colour highlights the HA of H5N1v from Indonesia. The node labels display the posterior value. The original sequences (GISAID ID) for worldwide HA of H5N1v phylogeny are displayed in Supplementary Table S1.

**Figure A1:**
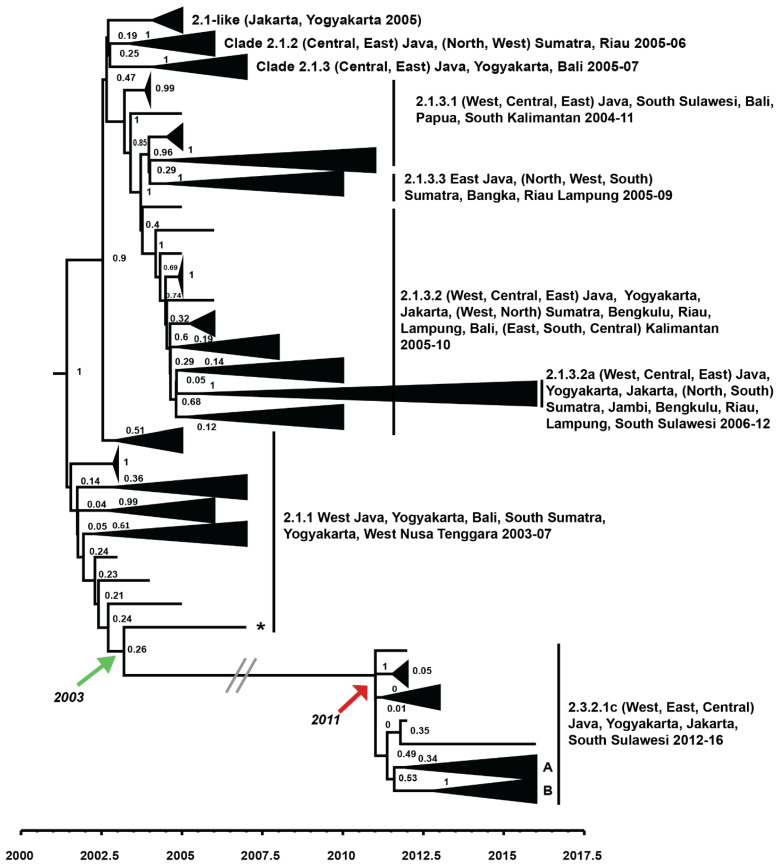
Time-measured phylogeny of HA genes of Indonesian poultry HPAIv A(H5N1) 2003–2016. The estimated root of HA clade 2.3.2.1c was highlighted by asterisk symbol, the tMRCA of HA clade 2.3.2.1c is pointed out by the red arrow, and the tMRCA between HA clade 2.1.1 and 2.3.2.1c is pointed out by the green arrow. The HPAIv A(H5N1) clade 2.3.2.1c diverged into subgroups (A and B). The node labels display the posterior value. The two gray lines between the clades 2.1.1 and 2.3.2.1c represent the presence of multiple viruses between these two clades as presented in Figure 1. The original sequences (GISAID ID) of HA genes of Indonesian poultry HPAIv A(H5N1) 2003–2016 phylogeny is displayed in Supplementary Table S1.

**Figure A2:**
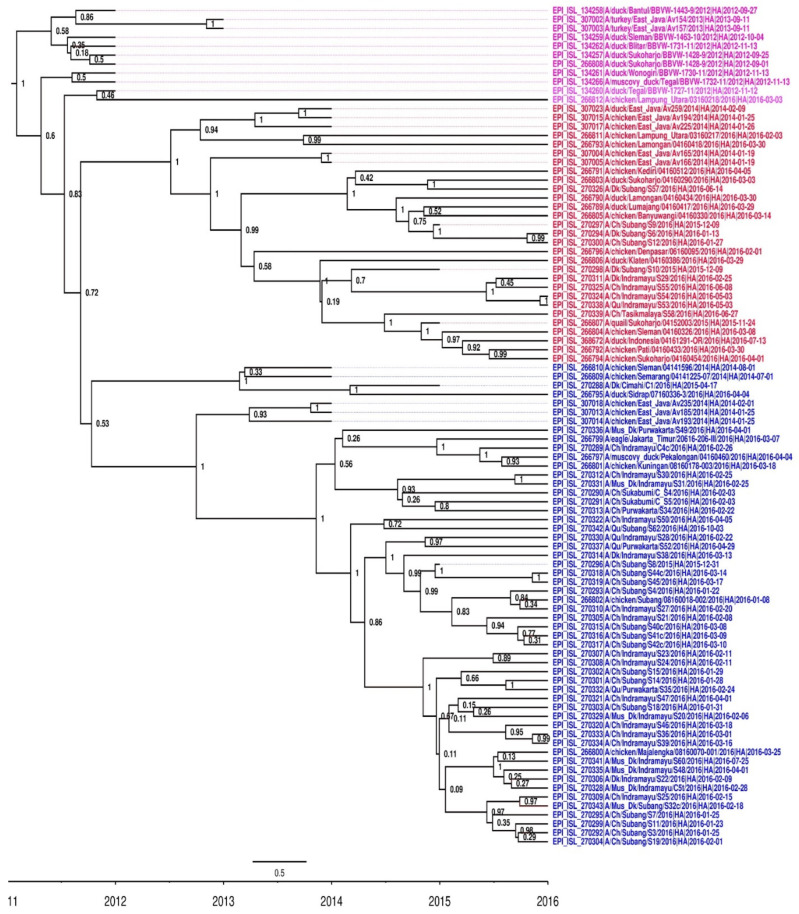
Time-scale phylogeny of Indonesian HA of H5N1v of HA genes of Indonesian poultry HPAIv A(H5N1) clade 2.3.2.1c. The clade 2.3.2.1c subgroup A is highlighted in red and clade 2.3.2.1c subgroup B is highlighted in blue. Another clade, 2.3.2.1c, is highlighted in pink. The node labels display the posterior value.

Time-measured phylogenetic analysis (Figure 1 and Figure A1) estimated that HPAIv A(H5N1) clade 2.1-like, 2.1.1, 2.1.2, 2.1.3, 2.1.3.1, 2.1.3.3, 2.1.3.2 and 2.1.3.2a evolved from a common ancestor in the year 2002. In addition, the analysis indicated that some of the HA clades 2.1.1 shared a common ancestor with 2.3.2.1c in the year 2001. Subsequently, the HPAIv A(H5N1) clade 2.1.1 in 2003 diverged into HPAIv A(H5N1) HA clade 2.3.2.1c, which was detected mostly in 2015/16. A significant divergence in 2011 was also observed between HA subgroup 2.3.2.1c (A) and 2.3.2.1c (B) (Figure A1) in the phylogenetic analysis, with posterior values of more than 0.7 (Figure A2).

In 2015–2016, HPAIv A(H5N1) clade 2.1.3.2a was still detected. In contrast, the clades 2.1-like 2.1.2, 2.1.3, 2.1.3.1, 2.1.3.2, and 2.1.3.3 were no longer detected in Indonesia after 2012. Detection of clades of HPAIv A (H5N1) in Indonesian poultry varied between years. From 2005 to 2007 (2 years), the HA clades 2.1.1, 2.1.2, and 2.1.3 were detected. Between 2005 and 2012 (7 years), the HPAIv H5N1 clade 2.1.3.1 was detected. From 2005 to 2010 (5 years), the virus HA sequences were classified as clades 2.1.3.2 and 2.1.3.3. Of the subclades of HPAIv A(H5N1) clade 2.1, only 2.1.3.2a was still detected in 2016, while subclade 2.3.2.1c was mostly detected after 2010.

**Figure 2:**
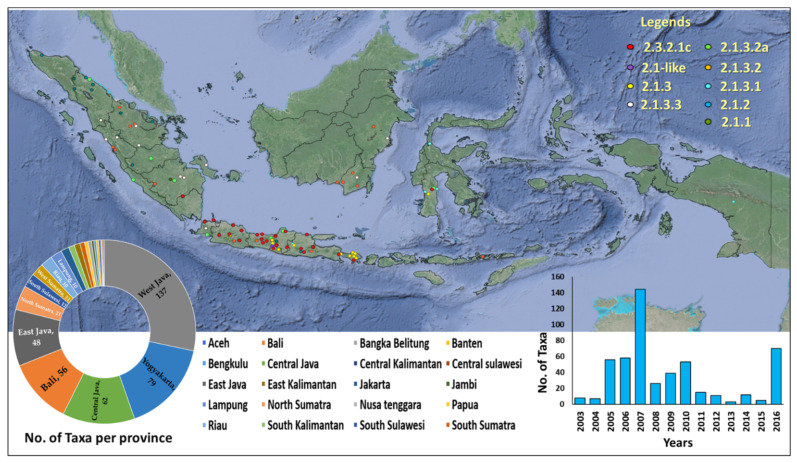
The distribution of HA of HPAIv A(H5N1) was detected in poultry based on different clades and different provinces of Indonesia. The number of taxa per province and per years are displayed in Supplementary Tables S2 and S3.

Spatiotemporal analysis indicated that various HA clades of Indonesian HPAIv A(H5N1) were detected in different areas (Figure 2). Most viruses were detected on Java Island. HA clades 2.1.3.2 and 2.1.3.2a were detected in most of the regions of Indonesia, while some clades were only detected in specific regions. For example, HPAIv A(H5N1) clade 2.1-like viruses were only detected in Jakarta and Yogyakarta, and HPAIv (AH5N1) clade 2.1.3 was only detected in Central Java, East Java, Yogyakarta, and Bali.

The BEAST analysis estimated that the mean nucleotide substitution rate of HA was 0.0042 substitution/site/year (s/s/y) (95% Interval, 0.0038–0.0046) over the course of 13 years. The mean substitution rate of clade 2.1.3.2a was 0.0042 (s/s/y) (95% Interval, 0.0031–0.0054) over an 8-year period, not statistically significant different than that of clade 2.3.2.1c (0.0036 s/s/y; 95% Interval, 0.0026–0.0041) over 4 years (Table 1).

### 3.2. Indonesian Viruses in the Phylodynamic of Indonesian Worldwide Avian Influenza H5N1 Virus (AI H5N1v) 

The phylogeny of the worldwide HA including the Indonesian viruses is depicted in Figure 1. Based on analysis of representative sequences, H5N1v clade 2.1 and its subclades were only detected in Indonesia, while clade 2.3 viruses were detected in multiple countries in Asia, Europe, and Africa, including Indonesia, since 2009. Other subclades of clade 2, such as 2.2, 2.4, and 2.5, were circulating in multiple countries, such as China, Egypt, Germany, India, and Japan, but were not detected in Indonesia. These different geographic distributions of viruses also indicate geographic imbalances in virus spread and geographic leaps of multiple viruses from various clades. 

The BEAST analysis estimated that the mean nucleotide substitution rate of global HA was 0.0065 substitution/site/year (s/s/y) over the course of 16 years (95% Interval, 0.0061–0.0070) (Table 1).

### 3.3. Molecular Dating of HPAIv A(H5N1) 

Results of molecular dating indicated that the common ancestor of HPAIv A(H5N1) detected in Indonesia occurred in May 2001, around 5 to 7 years after the common ancestor of HPAIv A(H5N1) worldwide. The common ancestor of HPAIv A(H5N1) clades 2.1.3.2. and 2.1.3.2a occurred in the first months of 2002 according to this analysis, while the common ancestor of clade 2.3.2.1c occurred in February 2011. Results of the tMRCAs of HA of HPAIv A(H5N1) detected in Indonesian poultry and worldwide, determined by using a relaxed clock, with 95% HPD and posterior values, are displayed in Table 2.

## 4. Discussion

### 4.1. Temporal Dynamic of Indonesian HPAIv A(H5N1): Time-Measured Phylogenetic Analysis 

In the present study, a time-measured phylogenetic analysis was performed to increase the understanding of the HPAIv A(H5N1) detected in Indonesia from 2003–2016. While phylogenetic analysis of HPAIv A(H5N1) was the focus of a number of studies already [11,22,32], a study including all available Indonesian virus sequences has, to our knowledge, not been performed previously. Posterior analysis of Indonesian HPAIv A(H5N1) 2003–2016 estimated that the HPAIv A(H5N1) clade 2.3.2.1 evolved from the HA clade 2.1.1. In addition, the posterior analyses using BEAST with bModeltest, instead of the maximum likelihood approach, which is used as a criterion in a unified nomenclature system for HPAIv, confirmed the finding of our previous study [11] that HA clade 2.3.2.1c consists of two different clusters [13,14,15]. The time-measured analysis also showed that after 2012, mainly HPAIv A(H5N1) viruses classified as clade 2.3.2.1c were detected. The observed evolution of HPAIv A(H5N1) viruses, the emergence of new clades, and the emergence of reassortments may have been caused by biosecurity gaps leading to reassortment and limited vaccine efficacy and poor vaccination coverage, although we cannot exclude circulation of these viruses in wild birds due to the very limited surveillance of avian influenza in wild birds in Indonesia [11,33,34,35].

The substitution rate of avian influenza viruses worldwide has been studied extensively [18,36,37]. A previous study [38] estimated viral RNA substitution rates in the range of 0.01 to 0.001 s/s/y. Additionally, the rapid evolutionary dynamics of avian influenza viruses were estimated by a previous study with a substitution rate range of 0.0018–0.0084 s/s/y [39]. The estimated substitution rate in this study showed the fast substitution rate of Indonesian poultry HPAIv A(H5N1) and HA of worldwide H5, which was in line with previous reports by Duffy et al. (10^–2^ to 10^–5^ subs/site/year) and Chen et al. (1.8 to 8.4 × 10^−3^ subs/site/year) [38,39], but different from those reported by Ducatez et al. (3.32 ± 0.05 × 10^−3^ subs/site/year) [40]. The variation in the substitution rates between the HPAIv A(H5N1) genes can be caused by many factors, such as the differences in viral biologies such as viral genome architecture, replication speeds within-host and viral polymerase enzyme fidelities [41], and environmental selectivity related to the host factors such as species [38], vaccination status [37], contact rate, and age of infection, epidemic, and endemic status in a region during infection [41]. Positive selection pressures related to environmental selectivity have been identified at several antigenic sites of the HA gene in the previous study [22]. Meanwhile, the mean substitution rate of global HA was higher than in Indonesian poultry HPAIv A(H5N1); this observation might, however, be biased by sampling differences.

The phylogenetic analysis estimated that HA clades 2.3.2.1a and 2.3.2.1c shared a common ancestor and were rooted in the clade 2.3.2.1b. The H5N1v clade 2.3.2.1c and 2.3.2.1a diverged from clade 2.3.2.1b in agreement with a previous study [13,15]. A gap in the H5N1v clades in Indonesia is indicated by the lack of report of clade 2.3.2.1b, the clade that has been reported in Vietnam and Hong Kong [15,42]. This clade gap was assumed based on the finding in Indonesia that the HPAIv A(H5N1) clade 2.3.2.1c was rooted in HA clade 2.1.1. Bird migration and/or poultry trade could have driven the transmission and evolution of the H5N1v clade 2.3.2.1a to clade 2.3.2.1c. Additionally, unrecognized clinical signs in poultry and the reluctance of farmers to report the H5N1 outbreaks, particularly in sector 1 farms, might have contributed to the absence of some clades of H5N1v in the data set. This gap shows the need for regular and intensive surveillance to control the evolution of H5N1v, not only in poultry but also in wild birds. 

The most recent ancestor of the H5N1 influenza virus in Indonesia has been previously studied [22,32]. The first study [22] estimated the tMRCA of Indonesian H5N1 HPAIV in June 2003 (November 2002 and October 2003) and the second study [32] estimated the tMRCA of reassortant H5N1v in July 2005. This study revealed that the common ancestor of Indonesian poultry HPAIv H5N1 was introduced into Indonesia 5–7 years (2001; 95% Interval: 1999–2002) after the original ancestor of HPAI A(H5Nx) arose worldwide (1996; 95% Interval: 1995–1996). The introduction of HPAIv A(H5N1) 5–7 years after worldwide outbreaks suggested the importance of sustainability of surveillance and control measures in around 5–7 years before the new introduction of new emerging and re-emerging HPAIv into Indonesia, either from outside Indonesia via wild birds or poultry trading of the virus, evolves within themselves in Indonesia.

### 4.2. Limitations and Benefits of the Study

We acknowledge several limitations in this study. First, the limited data, particularly the number of taxa or samples, may have affected the inferences of evolutionary analysis. Surveillance data and avian influenza virus sequences in wild birds in Indonesia are very limited or absent. All avian influenza sequence data in public genome databases were obtained from domesticated birds. Differences in sampling over time and space may affect the outcomes of the analysis. Therefore, improved surveillance with good competency for clinical and laboratory diagnosis and collection of metadata, as well as the willingness to share the information, is crucial to raise the number of viral genomes in the public database. Surveillance in wild birds is also crucial to reveal the clade gap and study the evolution of the avian influenza virus. Furthermore, additional studies are needed to identify key amino acid changes and evaluate their impact on the viral phenotype, and also on the relationship with the possible role of vaccination programs on the observed evolution of HPAIV A(H5N1).

This study is of importance not only for virus identification but also for studying virus evolution in Indonesia. This study shows that probably only two introductions occurred, after which HPAIv A(H5N1) continued to circulate among poultry in Indonesia. Continuous surveillance of poultry farms in all sectors and live bird markets in Indonesia with global support and collaboration are essential to take adequate measures and prevent further evolution of the virus. In addition, compartmentalization, inspection, and certification [43,44] of poultry farms are also important to control the evolution of HPAIv A(H5N1) in Indonesia. Estimation of temporal characteristics of HPAIv A(H5N1) across Indonesia in association with the viral dynamics is essential in conducting prevention controls such as quarantine, movement restriction, diagnostic tools, surveillance systems, and vaccine development [45,46] for future outbreaks. The discovery of different clades in only a few regions and the fact that some Indonesian HPAIv A(H5N1) clades were not detected in other countries indicates the importance of area- and country-specific preventive measures for HPAI outbreaks [45]. The Indonesian archipelago, with the ocean as a geographical barrier between islands and between continents, can be an advantage for the country and region-specific preventive measures, as well as reconstructions of intensive poultry farming locations and mapping of wild bird captive areas. In parallel, capacity building is of great importance for each country, and an agreed consensus between countries is a necessity in studying the viral phylodynamics, combined with regular genomic surveillance, to prevent future HPAIv pandemics. 

## 5. Conclusions

This study demonstrated that introductions of HPAIv A(H5N1) into Indonesia are infrequent and most of the observed changes in the virus originate from within Indonesia. The lack of detection of H5N1v clade 2.3.2.1b and the limited Indonesian HPAIv A(H5N1) genomic sequences in the database indicate that there is room for improvement in molecular surveillance of HPAIv in Indonesia. Furthermore, the evolutionary dynamics of the Indonesian HPAIv A(H5N1) highlight the need for continuing genomic surveillance and adequate control measures to prevent viral introduction and evolution, within and between farm transmission in different regions. 

## Figures and Tables

**Table 1:** The substitution rates of Indonesian HPAIv A(H5N1) 2003–2016 and representative worldwide HA of H5N1v with the estimation of root height.

Hemagglutinin Gene	Collection Time (Year)	Taxa (n)	Sites (Character)	Evolutionary Models	Substitution Rate (10^−3^ s/s/y)		Number of Substituted Sequences (Subst/Genome/Year)
					Mean	95% HPD Interval	
**Geographical Zone**							
Indonesian H5N1 (HA)	2003–2016	502	1559	TVM + Γ + I	0.0042	0.0038–0.0046	6
Global H5 (HA)	2005–2021	284	1723	TVM+ Γ + I	0.0065	0.0061–0.0070	11
**HA clade (Indonesian HA (H5N1))**							
Clade 2.1.3.2	2005–2010	203	1559	TIM1 + Γ + I	0.0046	0.0036–0.0056	7
Clade 2.1.3.2a	2008–2016	73	1730	TIM1 + Γ + I	0.0049	0.0032–0.0069	7
Clade 2.3.2.1c	2012–2016	94	1707	TIM1 + Γ + I	0.0036	0.0026–0.0046	6

The mean of rates is posteriorly estimated based on Bayesian MCMC analysis using evolutionary models. The number of sequences is labeled as a taxon (taxa). The character of the number of differed sites is normalized from the length of a sequence to get the proportion of differences between two sequences [24]. Abbreviations: TVM (transversion model), TIM (transition model), Γ (gamma), I (Invariant), bp (base pair), s/s/y (substitution/site/years).

**Table 2:** tMRCA of HA of HPAIv (H5N1) Indonesian poultry and worldwide H5, determined by using a relaxed clock, with 95% HPD and posterior values.

HA Gene	tMRCA	95% HPD Interval		Posterior
		Begin	End	
**Geographical zone**				
Indonesian H5N1 (HA)	27 May 2001	13 September 1999	2 July 2002	1.00
Global H5 (HA)	4 April 1996	27 May 1995	28 December 1996	1.00
**HA clade (Indonesian HA (H5N1)** **)**				
Clade 2.1.3.2	8 January 2002	27 May 1997	13 September 2004	1.00
Clade 2.1.3.2a	15 March 2002	2 July 1997	1 January 2006	1.00
Clade 2.3.2.1c	6 February 2011	13 September 2009	13 September 2011	1.00

## Data Availability

This study did not report any data.

## Supplementary Materials

The following supporting information can be downloaded at: https://www.mdpi.com/article/10.3390/v14102216/s1, Table S1: The GISAID ID of the HA sequences (taxa) for the Phylodynamic analysis in this study; Table S2: The number of Indonesian H5N1 taxa per location; Table S3: The number of Indonesian H5N1 taxa per year.
