# Supplementary material for: Phylodynamics of Highly Pathogenic Avian Influenza A(H5N1) Virus Circulating in Indonesian Poultry
Source: Viruses. 2022 Oct 8;14(10):2216. doi: 10.3390/v14102216 (PMC9608721; doi:10.3390/v14102216)
Supplement: Supplementary file 1 [file viruses-14-02216-s001.zip › viruses-1889649-supplementary.pdf]

Supplementary Materials for Phylodynamics of Highly Pathogenic Avian Influenza A(H5N1) virus circulating in Indonesian poultry

Table S1. The GISAID ID of the HA sequences (taxa) for the Phylodynamic analysis in this study

Sequences	GISAID ID
Indonesian HA	EPI_ISL_98578; EPI_ISL_98577; EPI_ISL_98576; EPI_ISL_98575; EPI_ISL_98574; EPI_ISL_98573; EPI_ISL_98572; EPI_ISL_98571; EPI_ISL_98570; EPI_ISL_98569; EPI_ISL_98568; EPI_ISL_98567; EPI_ISL_98566; EPI_ISL_98565; EPI_ISL_98564; EPI_ISL_98563; EPI_ISL_98562; EPI_ISL_98561; EPI_ISL_98560; EPI_ISL_98559; EPI_ISL_98558; EPI_ISL_98557; EPI_ISL_98556; EPI_ISL_93322; EPI_ISL_93253; EPI_ISL_93252; EPI_ISL_91624; EPI_ISL_91623; EPI_ISL_91553; EPI_ISL_91552; EPI_ISL_91551; EPI_ISL_91550; EPI_ISL_91549; EPI_ISL_91548; EPI_ISL_91547; EPI_ISL_91546; EPI_ISL_91545; EPI_ISL_91544; EPI_ISL_91543; EPI_ISL_91540; EPI_ISL_91539; EPI_ISL_91538; EPI_ISL_91537; EPI_ISL_91536; EPI_ISL_91535; EPI_ISL_91534; EPI_ISL_91533; EPI_ISL_91532; EPI_ISL_91531; EPI_ISL_91530; EPI_ISL_91529; EPI_ISL_91528; EPI_ISL_91527; EPI_ISL_91526; EPI_ISL_91524; EPI_ISL_91523; EPI_ISL_91522; EPI_ISL_91521; EPI_ISL_91520; EPI_ISL_91519; EPI_ISL_91518; EPI_ISL_91517; EPI_ISL_91516; EPI_ISL_91515; EPI_ISL_91514; EPI_ISL_91454; EPI_ISL_91453; EPI_ISL_91452; EPI_ISL_91451; EPI_ISL_91450; EPI_ISL_91449; EPI_ISL_91448; EPI_ISL_91447; EPI_ISL_91446; EPI_ISL_91445; EPI_ISL_91444; EPI_ISL_91443; EPI_ISL_91442; EPI_ISL_91441; EPI_ISL_91440; EPI_ISL_91439; EPI_ISL_91429; EPI_ISL_91428; EPI_ISL_91427; EPI_ISL_91426; EPI_ISL_91425; EPI_ISL_91424; EPI_ISL_91423; EPI_ISL_91422; EPI_ISL_91421; EPI_ISL_91420; EPI_ISL_91419; EPI_ISL_91418; EPI_ISL_91417; EPI_ISL_91416; EPI_ISL_91415; EPI_ISL_91414; EPI_ISL_91413; EPI_ISL_91412; EPI_ISL_91411; EPI_ISL_91410; EPI_ISL_91409; EPI_ISL_91408; EPI_ISL_91407; EPI_ISL_91406; EPI_ISL_91405; EPI_ISL_91404; EPI_ISL_91403; EPI_ISL_91402; EPI_ISL_91401; EPI_ISL_91400; EPI_ISL_91399; EPI_ISL_91398; EPI_ISL_91397; EPI_ISL_91396; EPI_ISL_91395; EPI_ISL_91394; EPI_ISL_91385; EPI_ISL_91384; EPI_ISL_91383; EPI_ISL_91382; EPI_ISL_91381; EPI_ISL_91380; EPI_ISL_91379; EPI_ISL_91378; EPI_ISL_91377; EPI_ISL_91376; EPI_ISL_91375; EPI_ISL_91374; EPI_ISL_91373; EPI_ISL_91372; EPI_ISL_91371; EPI_ISL_91370; EPI_ISL_91369; EPI_ISL_91368; EPI_ISL_91367; EPI_ISL_91366; EPI_ISL_91365; EPI_ISL_91364; EPI_ISL_91363; EPI_ISL_91362; EPI_ISL_91361; EPI_ISL_91360; EPI_ISL_91359; EPI_ISL_91358; EPI_ISL_91357; EPI_ISL_91356; EPI_ISL_91355; EPI_ISL_91354; EPI_ISL_91353; EPI_ISL_91352; EPI_ISL_91351; EPI_ISL_91350; EPI_ISL_91349; EPI_ISL_91348; EPI_ISL_91347; EPI_ISL_91346; EPI_ISL_91345; EPI_ISL_91344; EPI_ISL_91343; EPI_ISL_91342; EPI_ISL_87545; EPI_ISL_67389; EPI_ISL_67376; EPI_ISL_67375; EPI_ISL_67072; EPI_ISL_67067; EPI_ISL_67054; EPI_ISL_65649; EPI_ISL_65648; EPI_ISL_6486; EPI_ISL_64853; EPI_ISL_64842; EPI_ISL_64841; EPI_ISL_64840; EPI_ISL_64839; EPI_ISL_64837; EPI_ISL_64833; EPI_ISL_64826; EPI_ISL_64825; EPI_ISL_64824; EPI_ISL_64823; EPI_ISL_64820; EPI_ISL_64779; EPI_ISL_64631; EPI_ISL_64630; EPI_ISL_64629; EPI_ISL_64628; EPI_ISL_64627; EPI_ISL_64626; EPI_ISL_64625; EPI_ISL_64624; EPI_ISL_64623; EPI_ISL_64341; EPI_ISL_64340; EPI_ISL_64213; EPI_ISL_64174; EPI_ISL_64080; EPI_ISL_64079; EPI_ISL_64078; EPI_ISL_64077; EPI_ISL_64076; EPI_ISL_64075; EPI_ISL_64074; EPI_ISL_64073; EPI_ISL_64072; EPI_ISL_64071; EPI_ISL_64070; EPI_ISL_64069; EPI_ISL_5733; EPI_ISL_5732; EPI_ISL_368672; EPI_ISL_307023; EPI_ISL_307019; EPI_ISL_307018; EPI_ISL_307017; EPI_ISL_307015; EPI_ISL_307014; EPI_ISL_307013; EPI_ISL_307008; EPI_ISL_307005; EPI_ISL_307004; EPI_ISL_307003; EPI_ISL_307002; EPI_ISL_30656; EPI_ISL_30655; EPI_ISL_30654; EPI_ISL_30653; EPI_ISL_30652; EPI_ISL_30651; EPI_ISL_30650; EPI_ISL_30649; EPI_ISL_30648; EPI_ISL_30647; EPI_ISL_30646; EPI_ISL_30645; EPI_ISL_30644;

	EPI_ISL_30643; EPI_ISL_30642; EPI_ISL_30641; EPI_ISL_30640; EPI_ISL_30639; EPI_ISL_30637; EPI_ISL_30636; EPI_ISL_30634; EPI_ISL_30633; EPI_ISL_2917; EPI_ISL_284320; EPI_ISL_284317; EPI_ISL_284316; EPI_ISL_284315; EPI_ISL_270343; EPI_ISL_270342; EPI_ISL_270341; EPI_ISL_270339; EPI_ISL_270338; EPI_ISL_270337; EPI_ISL_270336; EPI_ISL_270335; EPI_ISL_270334; EPI_ISL_270333; EPI_ISL_270332; EPI_ISL_270331; EPI_ISL_270330; EPI_ISL_270329; EPI_ISL_270328; EPI_ISL_270326; EPI_ISL_270325; EPI_ISL_270324; EPI_ISL_270322; EPI_ISL_270321; EPI_ISL_270320; EPI_ISL_270319; EPI_ISL_270318; EPI_ISL_270317; EPI_ISL_270316; EPI_ISL_270315; EPI_ISL_270314; EPI_ISL_270313; EPI_ISL_270312; EPI_ISL_270311; EPI_ISL_270310; EPI_ISL_270309; EPI_ISL_270308; EPI_ISL_270307; EPI_ISL_270306; EPI_ISL_270305; EPI_ISL_270304; EPI_ISL_270303; EPI_ISL_270302; EPI_ISL_270301; EPI_ISL_270300; EPI_ISL_270299; EPI_ISL_270298; EPI_ISL_270297; EPI_ISL_270296; EPI_ISL_270295; EPI_ISL_270294; EPI_ISL_270293; EPI_ISL_270292; EPI_ISL_270291; EPI_ISL_270290; EPI_ISL_270289; EPI_ISL_270288; EPI_ISL_266812; EPI_ISL_266811; EPI_ISL_266810; EPI_ISL_266809; EPI_ISL_266808; EPI_ISL_266807; EPI_ISL_266806; EPI_ISL_266805; EPI_ISL_266804; EPI_ISL_266803; EPI_ISL_266802; EPI_ISL_266801; EPI_ISL_266800; EPI_ISL_266799; EPI_ISL_266798; EPI_ISL_266797; EPI_ISL_266796; EPI_ISL_266795; EPI_ISL_266794; EPI_ISL_266793; EPI_ISL_266792; EPI_ISL_266791; EPI_ISL_266790; EPI_ISL_266789; EPI_ISL_190642; EPI_ISL_190625; EPI_ISL_190619; EPI_ISL_190617; EPI_ISL_190614; EPI_ISL_190516; EPI_ISL_190515; EPI_ISL_167892; EPI_ISL_163459; EPI_ISL_163458; EPI_ISL_163457; EPI_ISL_163456; EPI_ISL_163455; EPI_ISL_163454; EPI_ISL_163453; EPI_ISL_163452; EPI_ISL_163451; EPI_ISL_163450; EPI_ISL_163449; EPI_ISL_163448; EPI_ISL_163447; EPI_ISL_163446; EPI_ISL_163444; EPI_ISL_163443; EPI_ISL_163442; EPI_ISL_163441; EPI_ISL_163440; EPI_ISL_163439; EPI_ISL_163438; EPI_ISL_163437; EPI_ISL_163436; EPI_ISL_163435; EPI_ISL_163434; EPI_ISL_163433; EPI_ISL_163432; EPI_ISL_163431; EPI_ISL_163430; EPI_ISL_163429; EPI_ISL_163428; EPI_ISL_163427; EPI_ISL_163426; EPI_ISL_163425; EPI_ISL_163424; EPI_ISL_163423; EPI_ISL_163422; EPI_ISL_163420; EPI_ISL_163419; EPI_ISL_163418; EPI_ISL_163417; EPI_ISL_163416; EPI_ISL_163415; EPI_ISL_163413; EPI_ISL_163412; EPI_ISL_163411; EPI_ISL_163410; EPI_ISL_163409; EPI_ISL_163408; EPI_ISL_163407; EPI_ISL_163406; EPI_ISL_163405; EPI_ISL_163404; EPI_ISL_163403; EPI_ISL_163401; EPI_ISL_163400; EPI_ISL_163399; EPI_ISL_163397; EPI_ISL_163396; EPI_ISL_163395; EPI_ISL_163394; EPI_ISL_163393; EPI_ISL_163392; EPI_ISL_163391; EPI_ISL_163390; EPI_ISL_163389; EPI_ISL_163388; EPI_ISL_163387; EPI_ISL_163386; EPI_ISL_163385; EPI_ISL_163384; EPI_ISL_163383; EPI_ISL_163382; EPI_ISL_163381; EPI_ISL_163380; EPI_ISL_163379; EPI_ISL_163378; EPI_ISL_163377; EPI_ISL_163376; EPI_ISL_163375; EPI_ISL_163374; EPI_ISL_163373; EPI_ISL_163372; EPI_ISL_163371; EPI_ISL_163370; EPI_ISL_163366; EPI_ISL_134266; EPI_ISL_134262; EPI_ISL_134261; EPI_ISL_134260; EPI_ISL_134259; EPI_ISL_134258; EPI_ISL_134257; EPI_ISL_13390; EPI_ISL_13388; EPI_ISL_13387; EPI_ISL_13386; EPI_ISL_13385; EPI_ISL_13384; EPI_ISL_13382; EPI_ISL_13381; EPI_ISL_13380; EPI_ISL_13379; EPI_ISL_13378; EPI_ISL_13377; EPI_ISL_13376; EPI_ISL_13374; EPI_ISL_13373; EPI_ISL_13372; EPI_ISL_13366; EPI_ISL_13349; EPI_ISL_13348; EPI_ISL_13345; EPI_ISL_13344; EPI_ISL_13343; EPI_ISL_13342; EPI_ISL_13341; EPI_ISL_13339; EPI_ISL_13334; EPI_ISL_13305; EPI_ISL_13304; EPI_ISL_13294; EPI_ISL_13293; EPI_ISL_13293; EPI_ISL_13292; EPI_ISL_13284; EPI_ISL_13283; EPI_ISL_13283; EPI_ISL_13282; EPI_ISL_13281; EPI_ISL_13275; EPI_ISL_13261; EPI_ISL_13260; EPI_ISL_13259; EPI_ISL_13258; EPI_ISL_13257; EPI_ISL_13256; EPI_ISL_11513; EPI_ISL_11513; EPI_ISL_11512; EPI_ISL_106095; EPI_ISL_106094; EPI_ISL_106089; EPI_ISL_106088;

	EPI_ISL_106087; EPI_ISL_106086; EPI_ISL_106084; EPI_ISL_106083; EPI_ISL_106082; EPI_ISL_106081; EPI_ISL_106073; EPI_ISL_106072; EPI_ISL_106071; EPI_ISL_106070; EPI_ISL_106069; EPI_ISL_106053; EPI_ISL_104854; EPI_ISL_104853; EPI_ISL_104852; EPI_ISL_104851; EPI_ISL_104850; EPI_ISL_104849; EPI_ISL_104848; EPI_ISL_104847; EPI_ISL_104056; EPI_ISL_10338;
Worldwide HA	EPI_ISL_10055; EPI_ISL_103197; EPI_ISL_10360; EPI_ISL_10378; EPI_ISL_104408; EPI_ISL_1081369; EPI_ISL_108410; EPI_ISL_108434; EPI_ISL_11362; EPI_ISL_1152; EPI_ISL_117763; EPI_ISL_117770; EPI_ISL_11887; EPI_ISL_119388; EPI_ISL_123278; EPI_ISL_123957; EPI_ISL_12401; EPI_ISL_12657; EPI_ISL_1290867; EPI_ISL_129354; EPI_ISL_129845; EPI_ISL_129889; EPI_ISL_130237; EPI_ISL_130380; EPI_ISL_13283; EPI_ISL_13345; EPI_ISL_135217; EPI_ISL_136245; EPI_ISL_137254; EPI_ISL_137553; EPI_ISL_139383; EPI_ISL_139384; EPI_ISL_139386; EPI_ISL_139508; EPI_ISL_13989; EPI_ISL_141284; EPI_ISL_14129; EPI_ISL_142593; EPI_ISL_143948; EPI_ISL_146766; EPI_ISL_148124; EPI_ISL_148130; EPI_ISL_148554; EPI_ISL_15159; EPI_ISL_151695; EPI_ISL_152086; EPI_ISL_15275; EPI_ISL_153620; EPI_ISL_15681; EPI_ISL_16190; EPI_ISL_162048; EPI_ISL_162422; EPI_ISL_167178; EPI_ISL_167374; EPI_ISL_170236; EPI_ISL_170538; EPI_ISL_170548; EPI_ISL_170549; EPI_ISL_170860; EPI_ISL_172815; EPI_ISL_173156; EPI_ISL_173687; EPI_ISL_174483; EPI_ISL_175618; EPI_ISL_180659; EPI_ISL_181417; EPI_ISL_190516; EPI_ISL_190617; EPI_ISL_190641; EPI_ISL_190642; EPI_ISL_193364; EPI_ISL_195600; EPI_ISL_195602; EPI_ISL_196570; EPI_ISL_198176; EPI_ISL_198771; EPI_ISL_198776; EPI_ISL_198876; EPI_ISL_198883; EPI_ISL_198916; EPI_ISL_199148; EPI_ISL_199271; EPI_ISL_199413; EPI_ISL_199444; EPI_ISL_201792; EPI_ISL_202557; EPI_ISL_205121; EPI_ISL_205503; EPI_ISL_205964; EPI_ISL_206302; EPI_ISL_208837; EPI_ISL_208842; EPI_ISL_21032; EPI_ISL_212420; EPI_ISL_219782; EPI_ISL_219845; EPI_ISL_219874; EPI_ISL_222478; EPI_ISL_229117; EPI_ISL_23104; EPI_ISL_231747; EPI_ISL_232043; EPI_ISL_232490; EPI_ISL_234280; EPI_ISL_234326; EPI_ISL_234370; EPI_ISL_234372; EPI_ISL_238009; EPI_ISL_241757; EPI_ISL_244526; EPI_ISL_244527; EPI_ISL_244533; EPI_ISL_245160; EPI_ISL_249691; EPI_ISL_252474; EPI_ISL_255754; EPI_ISL_256289; EPI_ISL_256291; EPI_ISL_256292; EPI_ISL_257010; EPI_ISL_257021; EPI_ISL_257072; EPI_ISL_257074; EPI_ISL_257078; EPI_ISL_257097; EPI_ISL_257449; EPI_ISL_257657; EPI_ISL_260058; EPI_ISL_262477; EPI_ISL_262481; EPI_ISL_266794; EPI_ISL_266798; EPI_ISL_266801; EPI_ISL_266825; EPI_ISL_268; EPI_ISL_273845; EPI_ISL_27608; EPI_ISL_277033; EPI_ISL_277041; EPI_ISL_278025; EPI_ISL_278504; EPI_ISL_278505; EPI_ISL_284317; EPI_ISL_285512; EPI_ISL_285622; EPI_ISL_28830; EPI_ISL_2917; EPI_ISL_2918; EPI_ISL_2920; EPI_ISL_295520; EPI_ISL_295793; EPI_ISL_295821; EPI_ISL_300660; EPI_ISL_30428; EPI_ISL_307001; EPI_ISL_307019; EPI_ISL_30875; EPI_ISL_321100; EPI_ISL_321130; EPI_ISL_333630; EPI_ISL_336925; EPI_ISL_337275; EPI_ISL_340826; EPI_ISL_340855; EPI_ISL_344530; EPI_ISL_348263; EPI_ISL_348277; EPI_ISL_354553; EPI_ISL_366635; EPI_ISL_376086; EPI_ISL_3770721; EPI_ISL_378628; EPI_ISL_379958; EPI_ISL_388007; EPI_ISL_388019; EPI_ISL_389117; EPI_ISL_389290; EPI_ISL_3895; EPI_ISL_4032280; EPI_ISL_4032309; EPI_ISL_4032542; EPI_ISL_4036; EPI_ISL_404985; EPI_ISL_404987; EPI_ISL_4055123; EPI_ISL_4055141; EPI_ISL_4055209; EPI_ISL_4055421; EPI_ISL_4055846; EPI_ISL_4061492; EPI_ISL_4061493; EPI_ISL_4062115; EPI_ISL_4062128; EPI_ISL_4069736; EPI_ISL_4071630; EPI_ISL_418178; EPI_ISL_4396748; EPI_ISL_4531; EPI_ISL_4533; EPI_ISL_4540; EPI_ISL_4580; EPI_ISL_502395; EPI_ISL_502397; EPI_ISL_503530; EPI_ISL_504924; EPI_ISL_61638; EPI_ISL_63001; EPI_ISL_63002; EPI_ISL_63018; EPI_ISL_63441; EPI_ISL_63499; EPI_ISL_63584; EPI_ISL_64847;

	EPI_ISL_6491; EPI_ISL_64915; EPI_ISL_65328; EPI_ISL_67383; EPI_ISL_67398; EPI_ISL_67753; EPI_ISL_6780544; EPI_ISL_6781351; EPI_ISL_6781353; EPI_ISL_6795258; EPI_ISL_6795270; EPI_ISL_68029; EPI_ISL_681286; EPI_ISL_6829533; EPI_ISL_697835; EPI_ISL_698060; EPI_ISL_707512; EPI_ISL_73303; EPI_ISL_74794; EPI_ISL_74796; EPI_ISL_74798; EPI_ISL_74799; EPI_ISL_75197; EPI_ISL_77984; EPI_ISL_77985; EPI_ISL_77996; EPI_ISL_77997; EPI_ISL_77998; EPI_ISL_77999; EPI_ISL_78001; EPI_ISL_78006; EPI_ISL_78013; EPI_ISL_78026; EPI_ISL_79686; EPI_ISL_79698; EPI_ISL_80477; EPI_ISL_80599; EPI_ISL_80605; EPI_ISL_80669; EPI_ISL_80684; EPI_ISL_80742; EPI_ISL_80744; EPI_ISL_80749; EPI_ISL_81468; EPI_ISL_81533; EPI_ISL_81534; EPI_ISL_81582; EPI_ISL_84854; EPI_ISL_8518268; EPI_ISL_8769034; EPI_ISL_91342; EPI_ISL_91349; EPI_ISL_91373; EPI_ISL_91520; EPI_ISL_91561; EPI_ISL_93253; EPI_ISL_93322; EPI_ISL_94038; EPI_ISL_94043; EPI_ISL_95061; EPI_ISL_95830; EPI_ISL_98194; EPI_ISL_98556; EPI_ISL_98559; EPI_ISL_98567; EPI_ISL_98568; EPI_ISL_98571; EPI_ISL_98574; EPI_ISL_98576; EPI_ISL_98577; EPI_ISL_98738;

Table S2. The number of Indonesian H5N1 taxa per location

Provinces	No. of Taxa
Aceh	2
Bali	57
Bangka Belitung	3
Banten	2
Bengkulu	2
Central Java	62
Central Kalimantan	1
Central Sulawesi	1
East Java	48
East Kalimantan	5
Indonesia	17
Jakarta	7
Jambi	2
Lampung	10
North Sumatra	21
Nusa Tenggara	1
Papua	3
Riau	10
South Kalimantan	5
South Sulawesi	12
South Sumatra	4
West Java	137
West Sumatra	11
Yogyakarta	79

Table S3. The number of Indonesian H5N1 taxa per year

Years	No. of Taxa
2003	8
2004	7
2005	55
2006	58
2007	144
2008	26
2009	38
2010	50
2011	15
2012	11
2013	3
2014	12
2015	3
2016	72
